# Genome-wide analysis of genetic variations between dominant and recessive NILs of glanded and glandless cottons

**DOI:** 10.1038/s41598-019-45454-y

**Published:** 2019-06-25

**Authors:** Tianlun Zhao, Cheng Li, Cong Li, Fan Zhang, Lei Mei, Elmon Chindudzi, Jinhong Chen, Shuijin Zhu

**Affiliations:** Department of Agronomy, Zhejiang University, Hangzhou, Zhejiang 310058 P.R. China

**Keywords:** Structural variation, Comparative genomics, Plant breeding, Polyploidy in plants

## Abstract

Cotton is an important economic crop in worldwide. It produces fiber for the textile industry and provides cottonseeds with high-quality protein and oil. However, the presence of gossypol limits the utilization of cottonseed. Two pairs of cotton near isogenic lines (NILs) with different pigment glands, i.e., Coker 312 vs Coker 312 W and CCRI12 vs CCRI12W, exhibit different gossypol contents. The glandless traits of Coker 312 W and CCRI12W are controlled by recessive and dominant genes, respectively. However, knowledge regarding the genomic variations in the NILs is limited. Therefore, the NILs genomes were resequenced and the sequencing depths were greater than 34×. Compared with the TM-1 genome, numerous SNPs, Indels, SVs, and CNVs were discovered. KEGG pathway analysis revealed that genes with SNPs and Indels from the recessive NILs and genes with Indels from the dominant NILs shared only one enriched pathway, i.e., the sesquiterpenoid and triterpenoid biosynthesis pathway, which is relevant to gossypol biosynthesis. Expression analysis revealed that key genes with variations that participate in the gossypol biosynthesis and pigment gland formation pathways had different expression patterns among the dominant, recessive glandless and glanded plants. The expression levels in the glanded organs were higher than those in their NILs. Altogether, our results provide deeper insight into cotton NILs with different pigment glands.

## Introduction

Cotton (*Gossypium* spp.) is a leading economic crop worldwide. In addition to producing nature fiber for the textile industry, cotton also provides a large quantity of cottonseeds containing high-quality protein and oil^1^. It is estimated that every kilogram fiber yield is associated with 1.65 kg cottonseeds, which contain approximately 21% oil and 23% protein^2^. However, the nutrient-rich cottonseeds cannot be used directly due to the presence of gossypol stored mainly in the pigment glands, which are toxic to human beings and monogastric animals^3^. Therefore, low-gossypol cotton cultivar breeding was always regarded as an important task. Two approaches may serve this purpose, i.e., either reducing the gossypol content directly or eliminating the pigment glands to reduce the gossypol content indirectly. Recently, some gossypol biosynthesis genes have been characterized and used to reduce the gossypol content in cottonseeds^2,4,5^. On the other side, as one of the most important secondary metabolites, gossypol plays important roles in cotton’s growth and development^6–8^, and is used as a medicine with anti-bacterial^9,10^, anti-cancer^11–13^ and male-contraceptive properties^14,15^.

The pigment glands, which are also called gossypol glands, appear as dark opaque dots in all parts of the glanded cotton plant^16^. The pigment glands are located on the surfaces of the ovaries, petals, sepals, bracts, stigma, stem and leaves. Anatomically the pigment glands are intercellular spaces with a large central cavity^17^, containing many gossypol and related terpenoids^6^. Previous research has reported that the gossypol content is highly correlated with the number of pigment glands^18^. Furthermore, it was proposed that different genotypes had an impact on the distribution of pigment glands^19^. Thus, the gossypol content in cotton plants is closely related to the genetic types of pigment glands in cotton cultivars. Therefore, investigating the formation of pigment glands is meaningful for regulating the content of gossypol in cotton plants.

The genetic mechanisms of pigment glands in cotton have been previously studied. The glandless cotton plant was thought to be mainly controlled by two recessive or one dominant gene. In previous research, two recessive genes, i.e., *gl*_2_ and *gl*_3_, were discovered to determine the glandless trait of the whole plant^20^, but these genes have not been characterized. In addition, the dominant gene controlling the glandless trait, i.e., $G{L}_{2}^{e}$ or *GoPGF*, was identified and cloned^21,22^. Furthermore, the specific mechanism of pigment gland formation is quite complicated and still unclear.

The next-generation sequencing technology has become more advanced in recent years. The genomes of *G. ramondii*^23^, *G. arboreum*^24^, *G. barbadense*^25^ and *G. hirsutum*^26^ have been sequenced. Therefore, the resequencing of some specific cotton accessions could provide deeper insight into the genomic variations in upland cotton. Based on the resequenced whole genomic variations between *G. hirsutum* and *G. barbadense* and between wild accessions and modern cultivars, the evolution and domestication history of allotetraploid cottons was revealed^27^. Based on a genomic analysis of 318 accessions, the signatures of selection and loci associated with fiber quality and yield traits were identified in cotton^28^. In addition, by genomic analysis of 352 accessions, a comprehensive variation map was constructed and 19 candidate loci responsible for fiber-quality-related traits were identified^29^. However, information regarding the genomic variation between a pair of specific trait near isogenic lines (NILs) such as glanded and glandless NILs is limited.

Coker 312 is an outstanding glanded upland cotton cultivar with a high content of gossypol, while Coker 312 W, which has a low gossypol content, is a recessive glandless near isogenic line (NIL) derived from Coker 312 by backcrossing, in which the glandless trait of Coker 312 W is controlled by two the recessive genes *gl*_*2*_ and *gl*_3_. Similarly, CCRI12W is a glandless NIL derived from CCRI12 by backcrossing, but the glandless trait is mainly controlled by one dominant gene named *GoPGF* or $G{L}_{2}^{e}$. Thus far, no data regarding the genomes of Coker 312, Coker 312 W, CCRI12 or CCRI12W have been reported. In this research, the whole genomes of these two pairs of NILs were resequenced by the next-generation sequencing platform to decipher the genomic variations. The results revealed genomic diversity as well as exposing the differences in several genes related to the corresponding traits between NILs, which could be useful for cotton breeding.

## Materials and Methods

### Plant materials and sampling

Two pairs of NILs, i.e., Coker 312 vs Coker 312 W and CCRI12 vs CCRI12W were used in the experiments. Z5629 is a glandless cultivar. and the glandless trait is controlled by a pair of recessive genes, i.e., *gl*2 and *gl*3. Coker 312 W was derived from a cross between Coker 312 and Z5629, and the glandless hybrid was selected from every generation of this cross for 16 generations. Hai-1 is a glandless cultivar, and the glandless trait is controlled by the dominant gene, $G{L}_{2}^{e}$. CCRI12W was derived from a cross between CRI12 and Hai-1, and backcrossed with CRI12 afterword for 16 generations. Coker312 and CCRI12 are glanded plants. The glandless trait of Coker 312 W is controlled by *gl2* and *gl3*, while CCRI12W is controlled by $G{L}_{2}^{e}$. The plants were grown in a greenhouse at Zhejiang University, Hangzhou, China. The leaves of the plants were sampled, frozen in liquid nitrogen and immediately stored at −70 °C.

### Image analysis

Slits of cottonseeds were examined under a stereo microscope (Leica MZ95, Germany) attached to a desktop computer. The images were captured by a stereo microscope.

### Extraction and determination of (±)-gossypol by High Performance Liquid Chromatography (HPLC)

The cottonseed samples were dried at 30 °C to constant weight for 2–3 days, and then ground with a grinder to a powder. Standard gossypol solutions were prepared by dissolving 0.01 g HPLC-grade gossypol in 10 mL of acetone. Standard gossypol solution of 0.010, 0.050, 0.100, 0.200, 0.500, 0.800, 1.000 and 2.000 mL were added into 10 mL volumetric flasks and adjusted to the calibration using acetone to prepare the different levels of gossypol solutions. Similarly, 0.1 g of sample was suspended into 2 mL acetone. In addition, the sample solutions were maintained in an ultrasonic bath for 45 min. Subsequently, the suspensions were filtered through quantitative filter paper followed by filtration with a 0.45 μm syringe filter (Agela, Newark, USA). The sediment was washed three times with acetone. Then, the extraction was adjusted to 10 mL with acetone.

The HPLC analysis was performed using an Agilent 1100 (Agilent, Santa Clara, USA), equipped with an auto-sampler and UV detection. A C18 column (Dikma, Richmond Hill, USA) (250 mm × 4.6 mm, 5 μm) was deployed as the stationary phase. The mobile phase consisted of Methanol/0.2% H_3_PO_4_ (80/20, v/v). The injection volume was 10 μL, and the flow rate was 1.0 mL/min. The UV detector was set at 238 nm, and the temperature was set at 25 °C. The samples were measured in triplicates.

### DNA extraction, library construction and sequencing

Genomic DNAs were extracted from the fresh leaves of the cotton plants using a standard cetyltrimethyl ammonium bromide (CTAB) protocol^30^. The DNA samples were randomly sheared to a size of ~350 bp. The DNA fragments were end-repaired, and an ‘A’ base was added to the end of the double strand break DNA. Then, DNA adaptors (Illumina, USA) were ligated to the above products. An agarose gel was used to separate these products and excised for purification. The modified and purified DNA fragments were enriched by PCR amplification with paired-end PCR primers (Illumina, USA). Qubit 2.0 (Life technologies, USA) was used to measure the concentration of the library. The libraries were diluted to 1 ng/μl, and the insert size of the libraries was detected by an Agilent Bioanalyzer 2100 (Agilent, USA). To ensure the quality of the libraries, quantitative RT-PCR (qRT-PCR) was used to accurately quantify the effective concentration of the libraries (>2 nM). Then, the libraries were sequenced on an Illumina HiSeq 2000 platform (Illumina, USA).

### Filtering and mapping reads

The paired-end reads were processed to remove adaptors and low quality paired reads for quality control and filtration. The procedure to remove the low quality reads were as follows: reads with adaptor; reads containing more than 10% ‘N’s; reads with more than 50% bases having low quality value (Phred score ≤ 5). The effective reads were aligned against the reference genome sequence of the upland standard line TM-1^26^ using Burrows-Wheeler Aligner (BWA)^31^. The compared results were calculated to remove the duplicated reads using SAMTOOLS^32^.

### Variations identification and annotation

MPILEUP in SAMTOOLS was used to identify the single nucleotide polymorphisms (SNPs) and insertions/deletions (Indels). In order to reduce the detection error rate of SNPs, the SNPs with the supported reads number less than four or quality value less than twenty were filtered. CREST^33^ was used to detect the structural variations (SVs) based on the alignment results and the insert size. CALL in CNVnator^34^ was used to detect the copy number variations (CNVs). Annotations of all the genetic variants were performed by ANNOVAR^35^. To visualize the genetic variants, Circos plots were generated by Circos^36^.

### QRT-PCR analysis

RNA samples were extracted from the samples using a Total RNA Extraction Kit (Aidlab, Beijing, China). The first strand cDNA was synthesized using a PrimeScript^TM^ 1st Strand cDNA Synthesis Kit (TaKaRa, Dalian, China). The primers used for qRT-PCR were designed with Primer 5.0 software. All primers are listed in Supplementary Dataset 1. The amplification reactions of qRT-PCR were performed with a Lightcycler 96 system (Roche) using SYBR the Premix Ex Taq (TaKaRa) with the following parameters: 30 seconds initializing denaturation at 95 °C, followed by 45 cycles of denaturation at 95 °C for 10 seconds; annealing at 53–54 °C for 10 seconds; and extension at 72 °C for 20 seconds. In addition, the default setting for the melting curve stage was chosen. The relative expression levels were calculated by the 2^−ΔΔCt^ method.

### Function annotation and classification of differential genes

Gene Ontology (GO) enrichment analysis of genes containing differential SNPs and Indels was implemented by the GOseq R package^37^. GO items with corrected *P-*values less than 0.05 were considered significantly enriched by different genes.

### Pathway analysis of differential genes

Kyoto Encyclopedia of Genes and Genomes (KEGG) pathway analysis of genes containing differential SNPs and Indels was performed by KOBAS software to test the statistical enrichment of different genes^38^. Pathways with corrected *P*-value less than 0.05 indicated that different genes were significantly enriched in those pathways.

### Accession codes

All the sequence data for two pairs of near isogenic lines have been deposited in the NCBI Sequence Read Archive under accession number PRJNA542238.

## Results

### Differences in pigment glands and gossypol contents among four upland cotton germplasms

**Figure 1:**
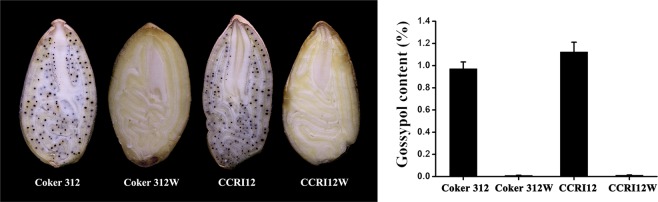
Cross sections and gossypol contents in Coker 312, Coker 312 W, CCRI12 and CCRI12W cottonseeds.

As the cottonseeds were crosscut, significant differences in the pigment glands were observed. The cottonseeds of Coker 312 and CCRI12 exhibited clearly visible pigment glands, while Coker 312 W and CCRI12W had no pigment glands (Fig. 1). Regarding the gossypol contents in the four cottonseeds, the gossypol contents in Coker 312 and CCRI12 were 0.973% and 1.125%, respectively (Fig. 1). However, the gossypol contents in Coker 312 W and CCRI12W were 0.011% and 0.014%, respectively, which were significantly lower than those in their glanded NILs.

### Sequencing results

The whole genomes of Coker 312, Coker 312 W, CCRI12 and CCRI12W were sequenced using Illumina HiSeq 2000, and 85.68, 84.28, 79.85 and 88.88 Gb raw data were output, respectively. After data filtration, 85.433, 83.971, 79.53 and 88.47 Gb clean data were obtained (Table 1). The results showed that the GC contents were 38.36 39.22, 39.42 and 38.88% in Coker 312, Coker 312 W, CCRI12 and CCRI12W, respectively. The four samples were all aligned to the reference genome sequence of TM-1 [23], and the mapping rates of the four samples were up to 99.64, 99.29, 99.22 and 99.68% (Table 2). The average genome coverage depths were both more than 34×.

**Table 1:** Statistics of the four germplasms sequencing.

Sample	Raw Base (bp)	Clean Base (bp)	Effective Rate (%)	Error Rate (%)	Q20 (%)	Q30 (%)	GC Content (%)
Coker312	85,686,024,300	85,433,099,700	99.70	0.03	96.45	91.47	38.36
Coker312W	84,282,812,700	83,971,926,000	99.63	0.03	96.44	91.36	39.22
CCRI12	79,846,896,900	79530271800	99.60	0.03	95.57	89.70	39.42
CCRI12W	88,879,908,600	88469693100	99.54	0.03	96.95	92.65	38.88

Summary of sequencing data quality.

**Table 2:** Statistics of the four germplasms mapping.

Sample	Mapped reads	Total reads	Mapping rate (%)	Average depth (X)	Coverage at least 1 × (%)	Coverage at least 4 × (%)
Coker312	567503992	569553998	99.64	36.27	97.18	91.89
Coker312W	555851656	559812840	99.29	35.88	96.09	90.04
CCRI12	526078285	530201812	99.22	34.01	96.88	90.09
CCRI12W	587925011	589797954	99.68	37.60	97.03	91.43

Summary of sequencing depth and coverage.

### SNPs and indels

The SNPs and Indels in Coker 312, Coker 312 W, CCRI12 and CCRI12W were obtained. SAMTOOLS^32^ and ANNOVAR^35^ were used for the detection and annotation of the SNPs and Indels.

**Figure 2:**
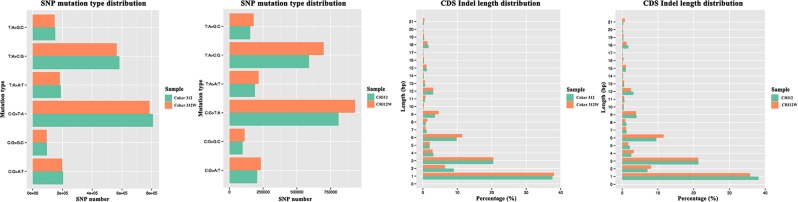
Distribution of SNPs and Indels. SNP mutation type distribution in Coker 312, Coker 312 W, CCRI12 and CCRI12W. CDS Indel length distribution in Coker 312, Coker 312 W, CCRI12 and CCRI12W.

A total of 2034021, 1974263, 2371614 and 2045734 SNPs were identified in the Coker 312, Coker 312 W, CCRI12 and CCRI12W genomes, respectively. According to the annotation results shown in Supplementary Dataset 2, 62101, 65761 73917 and 62389 SNPs are located in the exonic regions of Coker 312, Coker 312 W, CCRI12 and CCRI12W, respectively, while 32604, 35128, 39952 and 32760 SNPs of four genomes were non-synonymous. Moreover, there were 503, 552, 666 and 518 SNPs in Coker 312, Coker 312 W, CCRI12 and CCRI12W were stop gains causing the termination of gene expression, while 103, 121, 130 and 99 SNPs were stop losses causing the loss of the terminate codon, respectively. According to the statistical summary of all SNPs, six types of SNP mutations were found, which are presented in Fig. 2. Two types of changes including a T: A change to C: G and a C: G change to T: A contributed the most among the SNPs.

A total of 177324, 167973, 181706 and 180714 Indels, including insertions and deletions, were identified in the Coker 312, Coker 312 W, CCRI12 and CCRI12W genomes, respectively. According to the annotation results shown in Supplementary Dataset 2, 1203, 1267, 1525 and 1190 Indels were located in the exonic regions of Coker 312, Coker 312 W, CCRI12 and CCRI12W, respectively. In addition, among these Indels, 684, 689, 839 and 673 Indels were frameshift ones. Besides, 14, 18, 24 and 15 stop gains and 11, 9, 12 and 8 stop losses were found in the genomes. According to the statistical summary of all Indels, the distribution of the different length Indels in CDS regions is shown in Fig. 2. Indels with a length of 1 bp were the most common, followed by Indel with a length of three bp. In addition, the Indels with a long length only had contributed to a small proportion of total Indels.

Based on the genome comparison of Coker 312 vs Coker 312 W and CCRI12 vs CCRI12W, 1179465 and 1551376 differential SNPs were identified, respectively. All annotations of these SNPs are shown in Supplementary Fig. S1. In total, 27120 and 31737 differential SNPs existed in the exonic region, including synonymous, non-synonymous, stop gain, and stop loss SNPs in the two pairs of NILs, which might have an impact on the function or expression of their corresponding genes. However, most SNPs were located in the intergenic region.

A total of 136269 and 159682 differential Indels were also identified in the two pairs of NILs, i.e., Coker 312 vs Coker 312 W and CCRI12 vs CCRI12W, respectively. The annotation results of these Indels are also presented in Supplementary Fig. S1. There were 987 and 1297 differential Indels located in the exonic region, and the intergenic region Indels contributed the most as SNPs. Altogether, the distribution of SNPs and Indels with each pair of NILs on 26 chromosomes was shown in Supplementary Dataset 3.

### SVs and CNVs

SVs, such as insertions, deletions, inversions, intra-chromosomal translocations and inter-chromosomal translocations were detected and annotated by CREST^33^. CNVs, including deletions and duplications, were detected and annotated by CNVnator^34^.

**Figure 3:**
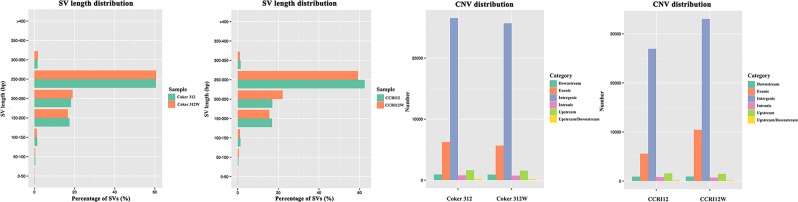
Distribution of CNVs and SVs. CNV annotation results and SV length distribution results in Coker 312, Coker 312 W, CCRI12 and CCRI12W.

A total of 3963, 3771, 4208 and 4026 SVs were identified in the Coker 312, Coker 312 W, CCRI12 and CCRI12W genomes, respectively. According to the annotation results presented in Supplementary Dataset 2, 237, 194, 244 and 203 SVs were located in the exonic regions of the four genomes. According to the statistical summary of all SVs, more than 55% of the total SVs had a length ranging from 250 to 300 bp (Fig. 3).

**Figure 4:**
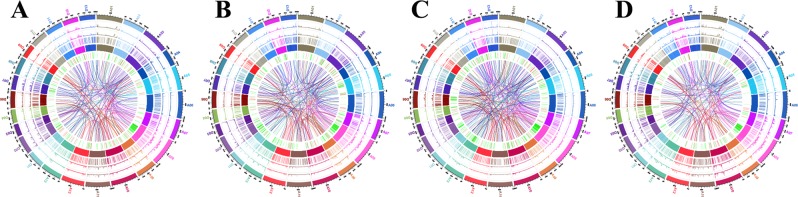
Circos plots of genome variations in Coker 312, Coker 312 W, CCRI12 and CCRI12W. (**A**) Coker 312; (**B**) Coker 312 W; (**C**) CCRI12; (**D**) CCRI12W. From the outside to the inside of the Circos plot, there are chromosomes, SNPs, InDels, CNV duplications, CNV deletions, SV insertions, SV deletions, SV invertions, SV intra-chromosomal translocations, and SV inter-chromosomal translocations.

A total of 36388, 34765, 46741 and 35976 CNVs were identified in the Coker 312, Coker 312 W, CCRI12 and CCRI12W genomes, respectively. In the recessive glandless NILs Coker 312 and Coker 312 W, there were 10054 and 10124 duplications with a total length of 137651200 and 162075400 bp, and 24641 and 26334 deletions with a total length of 358803300 and 349895000 bp, respectively. According to the annotation results shown in Supplementary Dataset 2, 6257 and 5656 CNVs were located in the exonic regions of Coker 312 and Coker 312 W, respectively. In the dominant glandless NILs CCRI12 and CCRI12W, there were 20843 and 9584 duplications with total lengths of 524737500 and 157062300 bp, and 25898 and 26392 deletions with total lengths of 424258300 and 361656500 bp, respectively. According to the annotation results shown in Supplementary Dataset 2, 10461 and 5587 CNVs were located in the exonic regions of CCRI12 and CCRI12W. The distribution of the CNVs is presented in Fig. 3. Circos plot of all variations in the genomes was displayed in Fig. 4, respectively.

### Functional significance of mutative genes with SNPs and Indels

A total of 18083 and 14913 genes with SNPs and 7172 and 8078 genes with Indels were identified after genomes comparisons of Coker 312 vs Coker 312 W and CCRI12 vs CCRI12W. GO enrichment and KEGG pathway analysis of these differential genes were carried out.

A GO enrichment analysis of these genes was conducted, and the thirty most significant GO items are plotted in Supplementary Fig. S2. This analysis showed that the genes with SNPs and Indels were distributed among the different gene ontologies. In the Coker 312 vs Coker 312 W comparison, the genes with SNPs were significantly enriched in three significant items in the molecular function ontology, including ADP binding, pattern binding and polysaccharide binding items. The genes with Indels were enriched in three ontologies including 18 items, such as nitrogen compound metabolic process, protein dimerization activity and cytoskeletal part. In the CCRI12 vs CCRI12W comparison, the genes with SNPs were significantly enriched in one biological process and two molecular function ontologies including cellular nitrogen compound metabolism process, terpene synthase activity and structural constituent of cell wall items. Among these ontologies, the terpene synthase activity item is closely related to gossypol biosynthesis. However, the genes with Indels had no significantly enriched items.

**Figure 5:**
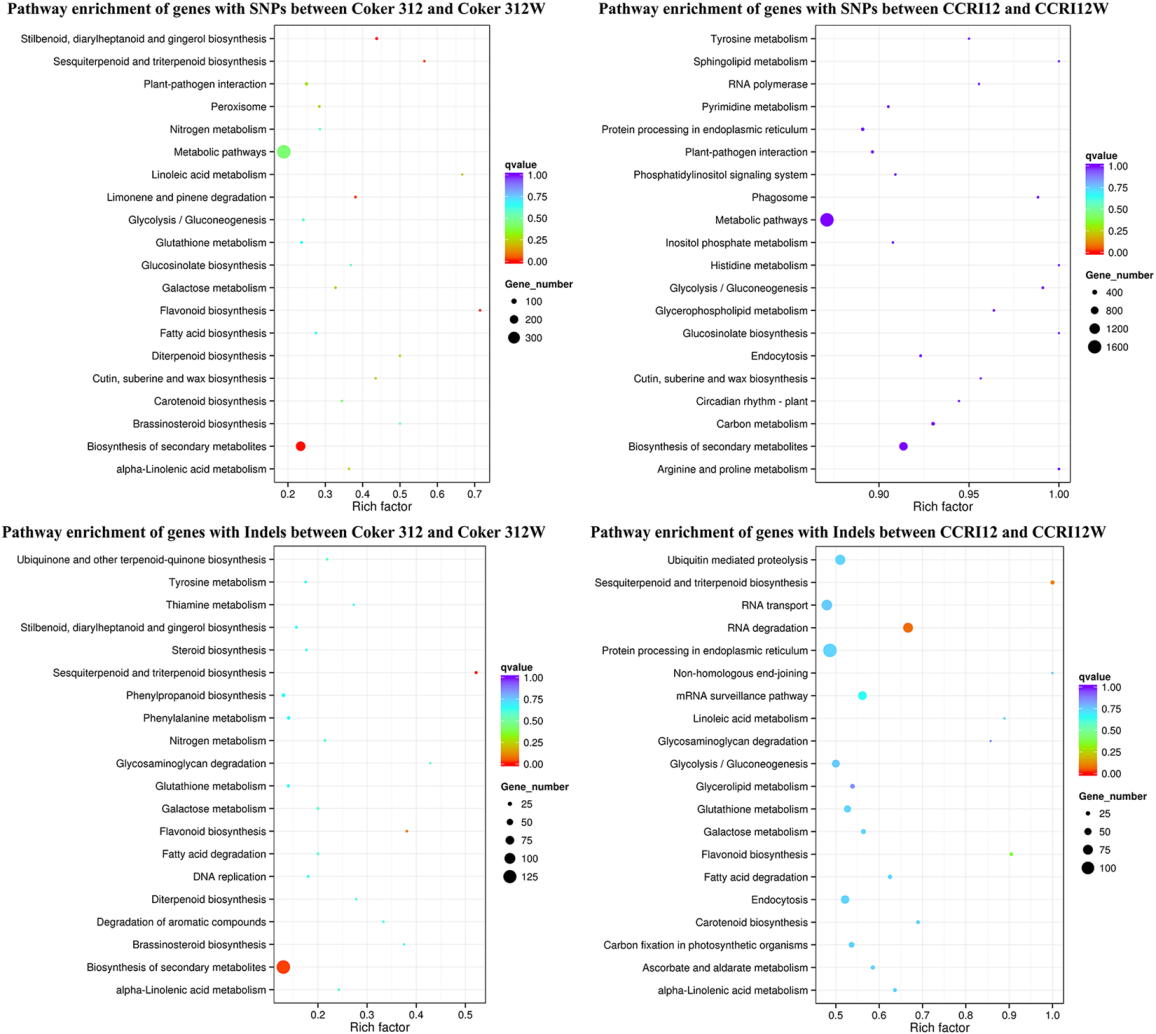
Pathway analysis enrichment results. Pathway analysis results of differential SNPs and Indels between Coker 312 vs Coker 312 W and between CCRI12 vs CCRI12W. *Indicated *P* < 0.05.

A KEGG pathway analysis of these differential genes was performed, and the most significant pathways are plotted in Fig. 5. In Coker 312 and Coker 312 W, the genes with SNPs were shown to be enriched in five pathways, including biosynthesis of secondary metabolites, flavonoid biosynthesis and sesquiterpenoid and triterpenoid biosynthesis. The genes with Indels were significantly enriched in two pathways, i.e., the sesquiterpenoid and triterpenoid biosynthesis pathway and biosynthesis of secondary metabolites pathway. In CCRI12 and CCRI12W, the genes with SNPs had no significantly enriched pathway. The genes with Indels were enriched in five pathways, including RNA degradation, sesquiterpenoid and triterpenoid biosynthesis pathway, flavonoid biosynthesis, etc. Through further comparison, it was found that the significantly enriched pathways of the SNPs and Indels in the dominant NILs shared two common pathways, i.e., the sesquiterpenoid and triterpenoid biosynthesis pathway and biosynthesis of secondary metabolites pathway. In addition, the genes with SNPs in the recessive NILs and the genes with Indels in the dominant NILs were found to share the flavonoid biosynthesis pathway. Furthermore, the genes with Indels in the dominant NILs and recessive NILs and the genes with SNPs in the recessive NILs were revealed to share the only one enriched pathway, i.e., sesquiterpenoid and triterpenoid biosynthesis pathway, which is closely related to the biosynthesis of gossypol. The genes enriched in the sesquiterpenoid and triterpenoid biosynthesis pathway were shown in Supplementary Dataset 4.

### Variations in key genes in gossypol biosynthesis and pigment gland formation, and qRT-PCR detection

In order to compare the genomes of Coker 312 vs Coker 312 W and CCRI12 vs CCRI12W, the key genes involved in the pathways of gossypol biosynthesis and pigment gland formation were selected to detect the genomic variations. The selected genes and identified variations are listed in Supplementary Dataset 5. Most variations in the SNPs, Indels and CNVs in all selected genes were located in the intergenic regions, while all SVs were located in the intergenic and exonic regions. In the Coker 312 vs Coker 312 W comparison, in the exonic regions, SNPs were found in the *TPS2, cad1-A, and GoPGF* genes; no Indels existed; SVs were discovered in *TPS1*, *hmg2*, *FPS*, and *WRKY1*; and CNVs only existed in *TPS2* and *cad1-A*. In the CCRI12 vs CCRI12W comparison, in the exonic regions, SNPs were found in *TPS1*, *hmg2*, *cad1-A*, and *GoPGF*; no Indel was existed; SVs were found in *hmg2*, *FPS*, and *WRKY1*; and CNVs existed in *TPS2*, *hmg2*, *cad1-A*, *CYP706B1*, and *WRKY1*.

**Figure 6:**
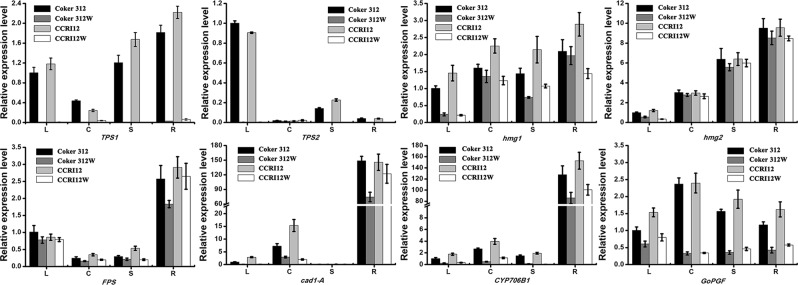
Gene expression profiles in Coker 312, Coker 312 W, CCRI12 and CCRI12W. L: leaf; C: cotyledon; S: stem; R: root. Values are reported as the means and standard deviation.

The genes participating in gossypol biosynthesis and pigment gland formation were differentially expressed as determined by a qRT-PCR analysis. From the upstream to the downstream of the gossypol biosynthesis pathway, we investigated the expression patterns of the key genes *TPS1, TPS2, hmg1, hmg2, FPS, CAD1-A, and CYP706B1*. As shown in Fig. 6, the terpene synthases genes *TPS1* and *TPS2* both had significantly higher expression levels in the four organs of the glanded plants than those in the glandless plants. *TPS1* and *TPS2* were almost not expressed in Coker 312 W and CCRI12W. Another two key genes, i.e., *hmg1* and *hmg2*, displayed a relatively higher expression level in the glanded cotton plants compared with those in the glandless cotton plants. In addition, the expression of *hmg1* greatly differed between the dominant NILs and recessive NILs. The expression of *hmg2* in the root was the highest, and the expression in the cotyledon was lower than that in the stem and higher than that in the leaf. The downstream genes in the gossypol biosynthesis pathway, i.e., *FPS*, *cad1-A*, and *CYP706B1*, had higher expression levels in all organs of Coker 312 and CCRI12 compared with those in Coker 312 W and CCRI12W. Furthermore, among the four organs, the expression level in root was significantly higher than those in the other organs regardless of whether the cotton plant was glanded or glandless, indicating that the root might be the main organ in the plant where gossypol is synthesized. The dominant pigment gland formation gene *GoPGF* also had a significantly higher expression level in the glanded plants compared with that in the glandless plants. Moreover, its expression in the cotyledon of the glanded cotton was higher than that in the other organs, while its expression in the cotyledon of the glandless cotton was lower than that in other organs, indicating that compared to other organs, the expression of *GoPGF* in the cotyledon greatly differs between glanded and glandless.

## Discussion

Cottonseed contains a large quantity of edible oil and high-quality protein and can attain the annual protein requirement of a half billion people^2^. However, the toxicity of gossypol and other related terpenoids stored in the pigment gland greatly hinder the effective utilization of cottonseed^3^. Recently, whole genome resequencing has been applied to many species for the detection of variations to research specific traits. Whole genome resequencing has been used to identify many alterations in radiation-induced red pericarp rice and predict eight genes involved in the biosynthesis pathways of beneficial products or pigment accumulation^39^. Similarly, the resequencing of the genomes of two strains revealed considerable genomic variations, and qRT-PCR analyses have determined that 26 effectors may be associated with phenotype and pathogenic differences^40^. Thus, in the present research, we constructed two pairs of NILs with different types of pigment glands, i.e., Coker 312 vs Coker 312 W and CCRI12 vs CCRI12W, and re-sequenced the whole genomes of these two pairs of NILs. The genomic differences were revealed and analyzed, and the expression of key genes related to gossypol biosynthesis and pigment gland formation was investigated.

Compared with the TM-1 genome, numerous SNPs, Indels, CNVs and SVs were revealed in the Coker 312, Coker 312 W, CCRI12 and CCRI12W genomes. Regarding the SNPs, the ratios of Non-synonymous/Synonymous SNPs in Coker 312, Coker 312 W, CCRI12 and CCRI12W were 1.13, 1.17, 1.13 and 1.20, respectively. In addition, there were many more transition SNPs than transversion SNPs, which is consistent with previous findings^41^. Regarding the Indels, single nucleotide insertions and deletions were the most common variations, which is consistent with previous reports in rice and soybean^42,43^. SVs and CNVs were detected and characterized for the first time in cotton, which could provide new sight into the variations in cotton genomes for better functional genome research.

The genome comparison among the NILs revealed many variations in the dominant NILs and recessive NILs genomes. The SNPs and Indels between Coker 312 and Coker 312 W were located in 14913 and 7172 genes, while SNPs and Indels between CCRI12 and CCRI12W existed in 18083 and 8078 genes, respectively. GO annotation was conducted, and the results showed that the genes with SNPs and Indels were significantly enriched in different ontologies, indicating that single nucleotide mutations and base insertion and deletion mutation occurred in different gene ontologies in one pair of NILs. Furthermore, the genes with SNPs in the recessive NILs and dominant NILs were significantly enriched in different items, while the genes with Indels in the recessive NILs enriched in 18 items, and the genes with Indels in the dominant NILs had no significantly enriched items. Therefore, the SNPs and Indels between the recessive and dominant NILs differed and the significantly enriched GO items also greatly varied. These findings suggest that the processes of gossypol biosynthesis and pigment gland formation might differ between recessive and dominant NILs.

Based on the KEGG analysis, in Coker 312 and Coker 312 W, the significantly enriched pathways with SNPs and Indels shared two pathways, i.e., the sesquiterpenoid and triterpenoid biosynthesis pathway and the biosynthesis of secondary metabolites pathway. The enriched biosynthesis of secondary metabolites pathway indicated that secondary metabolites might greatly differ between Coker 312 and Coker 312 W. In CCRI12 and CCRI12W, the genes with Indels were enriched in five pathways, including the sesquiterpenoid and triterpenoid biosynthesis pathway. Importantly, the commonly enriched sesquiterpenoid and triterpenoid biosynthesis pathway between the recessive NILs and dominant NILs was tightly connected to the traits of the different gossypol and relative terpenoid contents between the glanded and glandless cottons. Therefore, genes enriched in the sesquiterpenoid and triterpenoid biosynthesis pathway might be very significant for gossypol biosynthesis, which requires further research. These pathway analysis results indicated that the pathway of pigment gland formation was closely related to the related terpenoid biosynthesis. Therefore, conducting deeper research on the relationship between pigment gland formation and gossypol biosynthesis was meaningful. Moreover, flavonoid biosynthesis pathway was another commonly enriched pathway in the two NILs. Previous research had reported that flavonoid biosynthesis controlled the fiber color in naturally colored cotton by regulating the pigmentation process^44^. Therefore, the flavonoid biosynthesis pathway might be connected to the pigment gland traits differences, which also needs further investigation.

As gene expression can be affected by the position of SNPs and indels^42^, the variations in genes related to gossypol biosynthesis and pigment gland formation were investigated. SNPs, Indels, CNVs and SVs existed in many genes. From the upstream to the downstream of the gossypol biosynthesis pathway, the expression patterns of key genes, i.e., *TPS1, TPS2, hmg1, hmg2, FPS, CAD1-A, and CYP706B1* differed among the recessive and dominant glandless NILs. Previously, the terpene synthases genes *TPS1* and *TPS2* had been reported to be involved in plant defense^45^. *TPS1* had been reported to have a high expression level in the pericarp and a low expression level in the cotyledon^46^. In our research, *TPS1* also showed a low expression level in the cotyledon and a relatively high expression level in the stem and roots of the glanded plants, but *TPS1* showed almost no expression in the glandless NILs. *TPS2*, which is another monoterpene synthase gene, had been reported to have a high expression level in the leaf, a low expression level in the pericarp and nearly no expression in the other organs, and our results confirmed this gene characteristic. Interestingly, no expression was detected in Coker 312 W and CCRI12W, which might imply that terpene biosynthesis in glanded cotton plants is much stronger than that in glandless cotton plants and terpene biosynthesis in the cotyledon is weaker than that in other organs. Two key genes, i.e., *hmg1* and *hmg2*, encoding the hmg-coA enzyme are important for the gossypol biosynthesis pathway^47^. The expression of *hmg1* and *hmg2* in the roots was reportedly higher than that in the leaf. However, we further found that *hmg1* expression in the recessive NILs was higher than that in the dominant NILs, suggesting that the variation in *hmg1* might affect expression. Farnesyl diphosphate synthase is an important enzyme for the gossypol biosynthesis pathway^48^. The corresponding gene, i.e., *FPS*, is also involved in the resistance of cotton plant to *Veriticillium Dahlia*^49^. Here, we found high expression level of *FPS* in the root but a low expression level in the cotyledon regardless of whether the plant is glanded or glandless. *CAD1-A* is a (+)-*δ*-C synthase gene that is also significant for gossypol biosynthesis. Previously, *CAD1-A* had been reported to be highly expressed in glanded cottonseed, while the expression level in glandless cottonseed was meager^50^. Similarly, we observed that *CAD1-A* had a high expression in glanded cotyledon and a low expression in glandless cotyledon, but the roots of both glanded and glandless plants exhibit significantly high expression. Moreover, another critical gene participating in the gossypol biosynthesis pathway, i.e., *CYP706B1*, has good potential for the manipulation of the gossypol content in cottonseed^51^. This gene was also significantly highly expressed in root of glanded and glandless plants. Therefore, these three genes related to gossypol biosynthesis all had an extremely high expression level in the root of the glanded and glandless cotton plants, implying that the root might have a strong ability to synthesize gossypol and that the glandless cotton root could also effectively synthesize gossypol even though pigment glands are not located all over the plant. The only dominant pigment gland formation gene, i.e., *GoPGF*, has been characterized by Madan *et al*.^22^. We also investigated its expression patterns in four organs of the glanded plant and found that the expression of *GoPGF* in all organs of the glanded plant was higher than that in the glandless plant. *GoPGF* expression in the root of glanded plants was higher than that in the leaf and lower than that in the stem, which is similar to previous reports. However, we found that its specific expression patterns were quite different between the glanded and glandless plants. The relatively similar expression levels in the four organs in the glandless plants and the considerably different expression levels in the glanded plants may be due to the existence of pigment glands including the density and size of pigment glands. Therefore, the dominant gene in pigment gland formation exhibited a highly different expression pattern between the glanded and glandless cotton plants.

Low gossypol cotton cultivar breeding was always regarded as an essential task for breeders, especially for the development of glanded plants with glandless cottonseed. Therefore, research investigating pigment glands is also urgent. Nowadays, as genetic engineering technology developed, RNAi and CRISPR/Cas9 system were applied to research on the outstanding cotton variety breeding^2,52,53^. Sunilkumar *et al*. reported that they had successfully used RNAi to disrupt gossypol biosynthesis in cottonseed tissue by interfering with the *δ*-cadinene synthase gene expression. The present work based on the whole genomes of Coker 312, Coker 312 W, CCRI12 and CCRI12W provided lots of critical information for discovering genes controlling the biosynthesis of gossypol and the formation of pigment glands, which could be used as the candidate genes for genetic engineering breeding. With CRISPR/Cas9 system, we may use the discovered genomic information to develop the low-gossypol transgenic line and glandless transgenic line by knockout the critical genes in gossypol biosynthesis pathway, such as *CYP706B1*, and pigment gland formation pathway, such as $G{L}_{2}^{e}$, respectively. Furthermore, we may use seed-specific promoter with $G{L}_{2}^{e}$ gene to generate the cotton line with glanded plant and glandless cottonseed by RNAi or CRISPR/Cas9 system.

## Conclusions

Numerous DNA variations were identified in dominant and recessive glandless cotton NILs through re-sequencing technology. The comprehensive analysis of the variations revealed the different genes enriched in the ontologies and pathways related to the biosynthesis of secondary metabolites, which could provide a deeper insight into these four genomes. The expression profile of several genes related to gossypol biosynthesis and pigment gland formation revealed their significantly different expression pattern, which could be helpful for attaining a deep understanding of the mechanism of gossypol biosynthesis and pigment gland formation. Altogether, the large amount of sequenced data and related comprehensive analysis lay a foundation for uncovering the genes controlling pigment gland formation, which could be useful for cotton breeders developing new outstanding cotton germplasm with glandless seeds and glanded plants.

## Supplementary information

Supplementary Information

Supplementary Dataset
