# Supplementary material for: Genome-wide analysis of genetic variations between dominant and recessive NILs of glanded and glandless cottons
Source: Sci Rep. 2019 Jun 25;9:9226. doi: 10.1038/s41598-019-45454-y (PMC6593120; doi:10.1038/s41598-019-45454-y)
Supplement: Supplementary file 1 — Supplementary Information [file 41598_2019_45454_MOESM1_ESM.pdf]

Supplementary Information:

Genome-wide analysis of genetic variations between dominant and recessive NILs of glanded and glandless cottons

Tianlun Zhao, Cheng Li, Cong Li, Fan Zhang, Lei Mei, Elmon Chindudzi, Jinhong Chen & Shuijin Zhu*

Department of Agronomy, Zhejiang University, Hangzhou, Zhejiang, 310058, P. R. China. Correspondence and requests for materials should be addressed to S. Z. (email: shjzhu@zju.edu.cn)

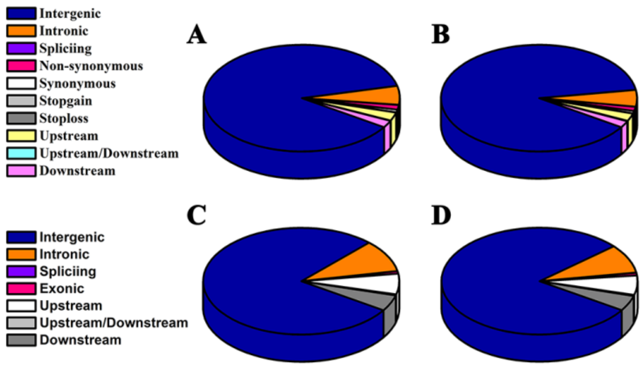

Supplementary Figure S1. Distribution of differential SNPs and Indels (A) SNP annotation results distribution between Coker 312 and Coker 312W. (B) Indel annotation results distribution between Coker 312 and Coker 312W. (C) SNP annotation results distribution between CCRI12 and CCRI12W. (D) Indel annotation results distribution between CCRI12 and CCRI12W.

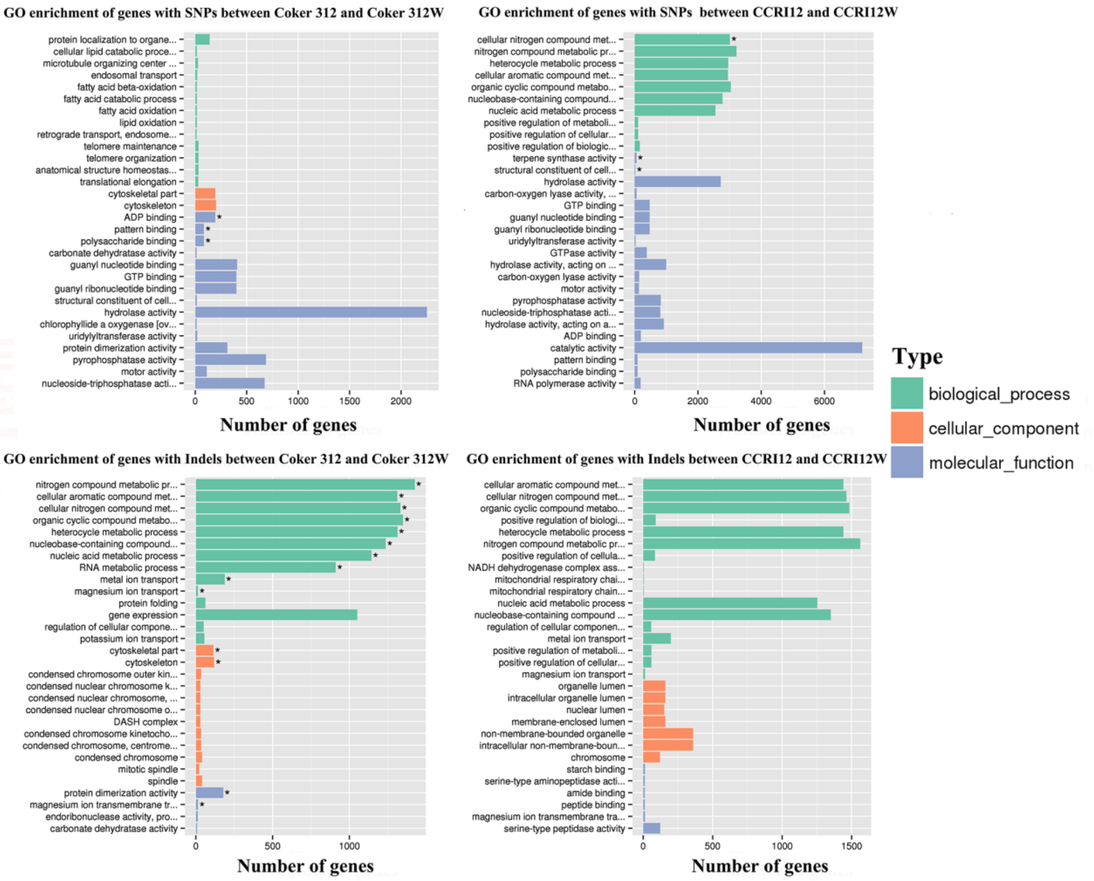

Supplementary Figure S2. GO analysis enrichment results. GO analysis results of differential SNPs and Indels between Coker 312 vs Coker 312W and between CCRI12 vs CCRI12W. * indicated $P < 0.05$.

Supplementary Dataset 1: All primers used for qRT-PCR

Supplementary Dataset 2: Summary of the annotations of the variations in Coker 312, Coker 312W, CCRI12 and CCRI12W

Supplementary Dataset 3: Distribution of differential SNPs and Indels within each pair of NILs in chromosomes

Supplementary Dataset 4: Genes enriched in the sesquiterpenoid and triterpenoid biosynthesis pathway

Supplementary Dataset 5: Variations in selected genes related to pigment gland formation and gossypol biosynthesis
